# *Mycobacterium bovis* in a Free-Ranging Black Rhinoceros, Kruger National Park, South Africa, 2016

**DOI:** 10.3201/eid2303.161622

**Published:** 2017-03

**Authors:** Michele A. Miller, Peter E. Buss, Paul D. van Helden, Sven D.C. Parsons

**Affiliations:** Stellenbosch University, Cape Town, South Africa (M.A. Miller, P.D. van Helden, S.D.C. Parsons);; South African National Parks, Skukuza, South Africa (P.E. Buss)

**Keywords:** black rhinoceros, bovine tuberculosis, *Diceros bicornis*, *Mycobacterium bovis*, South Africa, national parks, wildlife, tuberculosis and other mycobacteria, bacteria

## Abstract

In 2016, an emaciated black rhinoceros (*Diceros bicornis*) was found in Kruger National Park, South Africa. An interferon-γ response was detected against mycobacterial antigens, and lung tissue was positive for *Mycobacterium bovis.* This case highlights the risk that tuberculosis presents to rhinoceros in *M. bovis*–endemic areas.

Black rhinoceros (*Diceros bicornis*) are under severe threat from poaching and habitat loss. This species has been designated as critically endangered by the International Union for Conservation of Nature Red List (*1*). An estimated population of 5,000–5,445 animals are found in southern and eastern Africa, with just over 1,200 of those in South Africa (*2*). In Kruger National Park (KNP) in South Africa, the black rhinoceros population size is estimated at 400. KNP is considered an endemic area for *Mycobacterium bovis*, with cases reported in at least 12 wildlife species, including African buffalo, lion, kudu, and warthog (*3*).

Sporadic cases of tuberculosis (TB) caused by *M. tuberculosis* or *M. bovis* have been reported in black rhinoceros housed in zoos or under semi-intensive management (*4*). Although *M. bovis* is present in livestock and other wildlife species in countries in Africa where rhinoceros populations are currently present, no cases of TB have been reported in free-ranging black rhinoceros.

On June 17, 2016, rangers in KNP reported a weak, emaciated, adult female black rhinoceros that had been stationary for 36 hours in the southern area of the park (25°7′16′′S, 31°55′2′′E). The discovery of this animal might have resulted from increased surveillance related to poaching. When veterinary staff arrived, the rhinoceros was unresponsive and recumbent and lifted its head only when darted. External injuries were not obvious. Because of its poor prognosis, the animal was euthanized after being immobilized. Postmortem evaluation revealed an emaciated animal (body condition score 1 out of 5, http://www.daff.qld.gov.au/__data/assets/pdf_file/0015/53520/Animal-HD-Investigation-Condition-scores.pdf) with a subjectively heavy ectoparasite load. The subcutaneous and internal fat stores were reduced, consistent with the poor general body condition. Although teeth were worn, they appeared sufficient for mastication, and well-chewed ingesta was found in the gastrointestinal system. No grossly abnormal changes were found in the organs examined, except for the lungs and lymph nodes. On palpation of the lungs, numerous firm, focal, and irregular masses, 1–6 cm in diameter, were present in the right and left dorso-cranial two thirds of the lung lobes, with symmetric lesion distribution. On cut section, most lesions had a fibrous capsule and contained creamy necro-caseous material. Impression smears from the lung lesions revealed numerous acid-fast bacilli.

The heparinized whole blood samples that were collected before the animal was euthanized were incubated in Nil and TB Antigen tubes of the QuantiFERON TB Gold In-Tube system (QIAGEN, Venlo, Netherlands) and with pokeweed mitogen (Sigma-Aldrich Pty., Ltd., Johannesburg, South Africa) as a positive control. After 24 h, plasma was harvested and interferon-γ (IFN-γ) was measured in these samples by using a bovine IFN-γ ELISA (Mabtech AB, Nacka Strand, Sweden) as previously described for African buffaloes (*5*). IFN-γ concentrations measured in the sample from the Nil tube, pokeweed mitogen tube, and TB Antigen tube were 4 pg/mL, 753 pg/mL, and 175 pg/mL, respectively. The TB antigen-specific release of IFN-γ was consistent with immunologic sensitization to *M. bovis* or *M. tuberculosis* (*5*). We detected no antibodies to the *M. bovis* antigens MPB83 or ESAT6/CFP10 complex in serum samples tested with the Dual Path Platform VetTB assay (Chembio Diagnostic Systems, Inc., Medford, NY, USA) (*6*). PCR analyses confirmed *M. bovis* infection, both directly from the lung tissue and indirectly from mycobacteria culturing of lung tissue samples, as previously described (*7**,**8*).

Because black rhinoceros have been shown to be susceptible to TB, it is not unexpected to diagnose bovine TB in a free-ranging rhinoceros in an area with a high prevalence of TB in other wildlife species (*4*). Risk factors such as environmental load of mycobacteria, presence of concurrent disease, and other stressors (including malnutrition associated with drought) might result in progression of *M. bovis* infection. Although the source of infection for the animal we describe is unknown, no known exposure to humans or livestock has occurred. It is possible that interaction with other infected wildlife, including African buffalo, which are considered maintenance hosts of bovine TB, or environmental contamination at shared water holes and feeding sites might have resulted in pathogen contact (*8**,**9*). 

Occurrence of *M. bovis* infection in a free-ranging black rhinoceros in KNP might have substantial consequences for conservation programs. The risk for disease transmission between isolated, small populations of critically endangered species could hinder future translocation of these animals. Further risk assessments are needed to investigate the importance of this finding.
